# Recognising and Responding to Suicide-Risk Factors in Primary Care: A Scoping Review

**DOI:** 10.1007/s10935-024-00783-1

**Published:** 2024-05-27

**Authors:** Pooja Saini, Anna Hunt, Peter Blaney, Annie Murray

**Affiliations:** 1 School of Psychology, Faculty of Health, Suicide and Self-Harm Prevention, Liverpool John Moores University, Byrom Street, Liverpool, L3 3AF UK; 2 Department of Health and Social Care, Office for Health Improvement and Disparities, Piccadilly Place 3, Manchester, M1 3BN UK

**Keywords:** Suicide, Suicide prevention, Suicide risk, Primary care

## Abstract

The cost of one suicide is estimated to be £1.67 million (2 million euros) to the UK economy. Most people who die by suicide have seen a primary care practitioner (PCP) in the year prior to death. PCPs could aim to intervene before suicidal behaviours arise by addressing suicide-risk factors noted in primary care consultations, thereby preventing suicide and promoting health and wellbeing. This study aimed to conduct a rapid, systematic scoping review to explore how PCPs can effectively recognise and respond to suicide-risk factors. MedLine, CINAHL, PsycINFO, Web of Science and Cochrane Library databases were searched for three key concepts: suicide prevention, mental health and primary care. Two reviewers screened titles, abstracts and full papers independently against the eligibility criteria. Data synthesis was achieved by extracting and analysing study characteristics and findings. Forty-two studies met the eligibility criteria and were cited in this scoping review. Studies were published between 1990 and 2020 and were of good methodological quality. Six themes regarding suicide risk assessment in primary care were identified: Primary care consultations prior to suicide; Reasons for non-disclosure of suicidal behaviour; Screening for suicide risk; Training for primary care staff; Use of language by primary care staff; and, Difference in referral pathways from general practitioners or primary care practitioners. This review focused on better recognition and response to specific suicide-risk factors more widely such as poor mental health, substance misuse and long-term physical health conditions. Primary care is well placed to address the range of suicide-risk factors including biological, physical-health, psychological and socio-economic factors and therefore these findings could inform the development of person-centred approaches to be used in primary care.

## Introduction

Suicide is a major public health problem, both internationally and in the UK (World Health Organisation [WHO], 2023). Approximately 700,000 individuals die by suicide each year (WHO, 2023). *“The majority of suicides have been preceded by warning signs…it is important to understand what these warning signs are and to look out for them”* (WHO, 2014). The cost of one suicide is estimated to be £1.67 million (2 million euros) to the UK economy (Knapp et al., 2011). This includes indirect costs associated with the impact on those affected or bereaved by suicide.

Suicide prevention is recognised as a multisector public health obligation., and there is the importance of recognising where high-risk groups such as middle-aged males aged 45–49 years can be seen (John et al., 2020; HM Government, 2021). Suicide prevention should be addressed using collaborative methods with a shared objective in resolving a specific issue that can involve many ministries, government agencies, non governmental organisations, pertinent stakeholders, and other groups (WHO, 2023). The updated suicide prevention strategy for England (DH, 2023) highlights the important role of primary care for suicide prevention and three priority population groups for interventions: mental health patients, those who self-harm, and middle-aged men. These priority populations could potentially access support at different entry points within health, social care and community sectors e.g. mental health patients via mental health trusts; those who self-harm via ambulance services, emergency departments and educational settings; middle-aged men via workplace and community-based projects. All three groups could access their primary care practitioner (PCP) or practice nurse (PN) either through annual check-ups or ad-hoc appointments.

Public health is defined as the “art and science of preventing disease, prolonging life and promoting health through the organised efforts of society” (Acheson, 1988). Whilst traditionally, a Primary Care Practitioner’s (PCP; also known as general practitioners (GP) in the UK context) role was just to treat those who are sick, the National Health Service [NHS] now has a new focus on prevention. It is recognised that the NHS is unsustainable unless it has more of a prevention focus.

Suicide prevention in primary care is seen as a preventative opportunity (Michail et al., 2020; Mughal et al., 2021a) and this has become more important during the COVID-19 pandemic, which has been said to exacerbate mental health conditions (Mughal et al., 2021b). Since over 90% of people who die by suicide have seen a PCP the year prior (Haste et al., 1998; Luoma et al., 2002; Pearson et al., 2009; Rodi et al., 2010; John et al., 2020), PCPs could play a pivotal role in intervening before suicidal behaviours arise by addressing suicide-risk factors noted in primary care consultations, thereby preventing suicide and promoting health and wellbeing.

Common suicide risk factors include distal and proximal risk factors. Distal risk factors are background events and issues including a mental health diagnosis, previous suicide attempts and a family history of suicide or suicide attempts (Richardson et al., 2023). Proximal risk factors are more imminent warning signs including feelings of hopelessness, suicidal ideation, access to means, a major loss or stressful event and imprisonment (Sarkhel et al., 2023). Recognising imminent warning signs are important for primary care services as they are better positioned to treat proximal factors. Lower socioeconomic position is associated with an increased risk of suicidal behaviour (Knipe et al., 2017) and socio-economic factors such as deprivation, educational disadvantage, poor housing, and low income are prevalent across both distal and proximal risk factors. Usually, people who die by suicide have a combination of several distal and proximal suicide-risk factors.

The likelihood of dying by suicide varies across a range of populations based on sex, age, sexuality, socio-economic status and occupation (Knipe et al., 2017). For example, in most countries suicide rates are highest among men but in some Asian countries there is a variation or rates across sex (Jordans et al., 2014). Many of these factors are also linked to poor physical health (e.g. chronic conditions) and poor mental health. There is a common misconception that suicide is only an issue for those experiencing poor mental health. However, only 28% of general population suicides were in contact with mental health services in the year prior to death (Appleby et al., 2018); and not everyone who dies by suicide has experienced poor mental health. Addressing health inequalities and premature deaths are key policy drivers and preventing suicides in populations with an increased likelihood of dying by suicide is vital.

Primary care is a key setting in any suicide prevention programme as 31% of those who died by suicide saw their GP in the preceding month before their death (John et al., 2020) and as previously mentioned those in touch with mental health services in the year prior to death were even more likely to have seen a GP (90%; Pearson et al., 2009). The WHO (2014; 2023) describes three levels of suicide prevention programmes and suggests possible interventions,

1. “Universal” programmes aimed at reaching the whole population
2. “Selective” programmes that target populations at increased risk i.e. those who have experienced one or several suicide-risk factors
3. “Indicated” programmes that target specific populations at increased risk e.g. those who self-harm, middle-aged men and mental health patients

Interventions highlighted by the WHO (2014; 2023) that are relevant for PCPs include improved access to health care, promotion of good mental health, addressing substance misuse, limiting access to the means of suicide, “gatekeeper” training; and better recognition and response to specific suicide-risk factors such as poor mental health, substance misuse and long-term physical health conditions (WHO, 2014; 2023). Previous reviews have been conducted that focus on suicide prevention for high-risk groups, assessment of suicide prevention in primary care and evidenced-based suicide prevention interventions (O'Connor et al. 2013; Milner et al., 2017; Dueweke et al., 2018; Mann et al., 2021; Spottswood et al., 2022), however they have not focused on better recognition and response to specific suicide-risk factors more widely such as poor mental health, substance misuse and long-term physical health conditions. The aim of this scoping review is to understand how PCPs can effectively recognise and respond to suicide-risk factors in a way that keeps patients attending in primary care safe and reduces the outcome of suicide.

## Methods

### Design

A scoping review design (Arksey & O’Malley, 2005; Levec et al., 2010) was used to identify and explore literature examining suicide prevention and recognition of risk factors in primary care. Although a systematic review was considered, due to the emergent nature of the research topic and diverse study designs in the area, a scoping review was thought to be optimal at this stage to explore a broad range of studies.

Two authors (AH, PB) independently reviewed titles, abstracts and full texts against the eligibility criteria. Discrepancies regarding the inclusion or exclusion of studies was resolved though discussion with a third reviewer (PS). There was high agreement between the two authors (AH, PB; 85%) on which studies were to be included or excluded for this scoping review.

**Table 1:** Scoping Search Terms

Suicide		Additional terms
Suicide OR Suicide Prevention OR Suicide Risk	AND	Primary Care OR Mental Health

Data was extracted from studies into a custom-made table capturing author, year, title and key themes (Table 2).

**Table 2:** Quantitative Studies

Author/year/location	Purpose	Participants	Study design	Results
Anderson et al (2015) United Kingdom	To review how often suicide ideation and attempts is recorded in Electronic Health Records (HER)	61,464 EHR data sets from a Primary Care provider	Retrospective analysis of data	Only 3% of patients with an indication of suicidal ideation had a record in their EHR
Appleby et al (1996) United Kingdom	To review GP attendance in young men prior to suicide	61 males under 35 years who died by suicide	Retrospective analysis of data	The number of GP visits increased significantly before death
Bajaj et al (2008) United Kingdom	To assess attitudes to screening for suicidal ideation	101 patients diagnosed with depression 103 GPs at three practices across in North West London	Experimental Design	A majority of both GPs and patients stated that patients should be screened for suicidal ideation
Binder et al (2020) Multi-Europe	To assess the use of the BITS questionnaires in identifying suicidality in adolescents	693 patients aged 13–18	Experimental design	The BITS questionnaire was a useful tool in identifying suicidal risk regardless of reason for consultation
Crawford et al (2011) United Kingdom	To examine whether screening for suicidal ideation among people who attend primary care services and have signs of depression increases the short-term incidence of feeling that life is not worth living	443 patients across four general practices who showed signs of depression	Randomised control trial	No evidence was found that screening for suicidal ideation increased feelings that life is not worth living. More than 1 in 8 participants reported thoughts of taking their own life in the previous 2 weeks
Diamond et al (2017) United Kingdom	To identify profiles of risk behaviours and social stress associated with suicidal ideation and behaviour using the Behavioural Health Screen	Screening data from 2513 primary care patients aged 14–24 years	Retrospective analysis of data	High and low risk factor profiles were identified. The high-risk group was 11 times more likely to have made a suicide attempt, five times more likely to report a history of suicidal ideation and behaviour, and three times more likely to report recent suicidal ideation and behaviour
Dube et al (2010) USA	Evaluate evidence for the P4 screener as a brief measurement tool to assess potential suicide risk	250 Primary Care patients with clinical depression and comorbid chronic musculoskeletal pain	Randomised Control Trial	The P4 screener may be useful in assessing potential suicide risk in the clinical care of depressed patients
Dueweke et al (2018) USA	Review the use of the PHQ-2 for identifying suicidal patients	548 adult Primary Care Patients	Retrospective analysis of data	The PHQ-2 did not improve explanation of suicidal thoughts. Additional practitioner questions should be asked
Finnegan et al (2018) USA	The study aimed to evaluate and identify barriers to effective suicide risk assessment	31 Primary Care Providers	A cross sectional survey	There should be enhanced training in this area to make PCPs more comfortable in assessing suicide risk
Frankenfield et al (2000) USA	To assess the screening of adolescents for suicidal behaviour by primary care providers	693 Primary care providers	A cross sectional survey	Most practitioners did not routinely screen for suicidal thoughts or behaviours and more training is needed in this area
Haste et al (1998) United Kingdom	To identify, in suicide cases and matched controls, the patterns of consultation, diagnosis, and treatment of mental illness, and recording of risk factors for suicide	339 suicide cases and 3 matched controls for each case	Retrospective analysis of data	Females consulted with GPs more frequently than males, although the majority of cases (80%) were male
Heisel et al (2010) USA	To assess if the GDS scale is suitable for screening for suicidal risk in older adults	626 adults aged 65+	A cross sectional survey	The GDS accurately identified suicidal risk in older adults
Hooper et al (2012) USA	To review the factors related to a PCP enquiring about suicidal risk in a consultation	404 Primary Care Physicians	A cross sectional study	Although 36% of PCPs enquired about suicide the majority did not. More training is required in this area
Jerant et al (2020) USA	To examine the effect of a tailored interactive computer program (MAPS) designed to encourage middle-aged men’s discussion of suicide with PCP	48 men aged 35+ who had expressed thoughts of suicide	Randomized control trial	When compared with a control group, MAPS encouraged men with suicidal thoughts to discuss these feelings with their PCP
Kaplan et al (1999) USA	To review the differences by speciality of physician when supporting an older patient with depression and/or suicidality	300 primary care physicians	A cross sectional survey	Significant differences in treatment procedures, assessments and referrals was observed across specialities
Mesec et al. (2010) Slovenia	To review last Primary Care visit for patients who died by suicide	77 case files of people who had died by suicide from 1993 to 2003	Retrospective analysis of data	Compared to 3% of controls, 30% of patients who died by suicide last visited their primary care physician regarding their mental health
Milne et al (1994) United Kingdom	To review the amount of psychiatric and physical morbidity in people who had died by suicide	665 case files of people who had died by suicide from 1988 to 1989	Retrospective analysis of data	Mental illness was not identified in a significant number of suicide victims, whilst chronic pain and illness was identified as a risk factor
Parisi et al (2019) United Kingdom	To investigate psychiatric comorbidity, medication prescribing and risk of suicidality in people with psoriasis	56,961 with psoriasis 876,919 without psoriasis	Retrospective analysis of data	Suicide risk was lower in people with psoriasis diagnosed at 40+ years
Pfaff et al. (2005) Australia	To review to prevalence of suicidal ideation in patients aged 60 +	1061 patients aged 60 +	Cross sectional survey	25% of patients had signs of psychological distress and / or suicidal thoughts
Pigeon et al (2019) USA	To assess the effects of a brief CBT for insomnia course for primary care patients diagnosed with depression or PTSD	54 primary care patients diagnosed with depression or PTSD	Randomized Control Trial	The CBT for insomnia course has the capacity to improve mood and sleep in patients endorsing suicidal ideation. However it should be used in parallel with other support
Power et al (1997) United Kingdom	To review the characteristics of those who died by suicide compared with a matched control group	41 patient case notes who died by suicide	Retrospective analysis of data	Those who died by suicide visited their GP more frequently than the control group over a 10 year period, but not in the month before their death
Robles et al (2019) Mexico	To examine the impact of a training course for primary care physicians focusing on the identification of depression and suicide risk	60 primary care physicians	Experimental design	A brief training course could improve primary care physicians ability to identify and manage suicide risk
Roškar et al (2010) Slovenia	To evaluate an educational programme aimed at primary care physicians on the recognition and treatment of depression and suicide prevention	82 primary care physicians	Experimental Design	After the programme there was an increase in the prescription of antidepressants in the area. However, there was no significant reduction in suicide rates
Schou et al. (2019) Denmark	To assess the utilization of primary care in the year proceeding suicide	11,191 cases of people who died by suicide from 1997 to 2013	Retrospective analysis of data	Almost 70% of patients did not attend primary care in the month before their suicide
Stanistreet et al (2004) United Kingdom	To compare data on the timing and nature of final GP contacts before death among young men	172 patient case notes/inquest reports (80 of which were ruled suicide)	Retrospective analysis of data	Patients who died by suicide were more likely to have seen a mental health professional prior to death. Very few men who died by suicide consulted with their GP prior to death
Vannoy et al (2010) USA	To review the language and context used when discussing suicide with a patient	152 primary care physicians	Secondary data analysis	Although the majority of language was used was clear, some primary care physicians unintentionally reinforced the idea of patients not revealing suicidal thoughts
Vannoy et al. (2011) USA	To characterise discussion of suicide with patients diagnosed with depression	48 primary care physicians 1776 patients	Secondary data analysis	Suicide was often not addressed with depressed patients and often with those who presented the lowest risk
Windfuhr et al (2016) United Kingdom	To examine suicide risk in relation to patterns of GP consultation, psychotropic drug prescribing and psychiatric diagnoses	2384 patients aged 16+ who died by suicide	Retrospective analysis of data	Patients who did not consult with a GP were at increased risk of suicide suggesting current primary care methods need to be addressed
Younes et al (2013) France	Compare characteristics of young adult (18–39 years) suicide attempts with those of older adults (40 + years)	270 patient case notes	Retrospective analysis of data	In comparison with older adults, young adults consulted their GP less frequently in the month preceding the suicidal attempt
Younes et al (2020) France	To review primary care management before and after a suicide attempt	321 patient case notes/reports between 2013 and 2017	Retrospective analysis of data	GPs are involved in the management of patients at the time of a suicide attempt for 30% of patients. 50% of patients consulted with their GP afterwards

The research question was ‘How can primary care practitioners effectively recognise and respond to suicide-risk factors?’.

### Search Strategy

Five electronic databases (MedLine, CINAHL, PsycINFO, Web of Science and Cochrane Library databases) were searched through the dates 1990 to 2020. A combination of search terms were identified to produce the most relevant results. Identification of relevant studies were conducted independently by two researchers (see Table 1).

Whilst there is overlap between the bibliographic databases Medline and Embase, both were searched to ensure the research evidence and theories of suicide included European as well as United States of America (USA) research. Medline tends to have a greater focus on USA publications whilst Embase is more euro-centric (which in this case may be more relevant as the USA does not offer free health care to all). Eligible full texts were subjected to data extraction by the two of the suthors (XX, XX). Data were extracted on the study aims, design, location, sample size and demographic information. Studies considered were limited to those written in English. Studies were excluded if they were a review, a report based on secondary data, were on assisted suicide and when they did not have a focus on primary care and suicide.

Date limits were placed on the terms “suicide”, “suicide prevention”, “suicide risk” “mental health” and “primary care”. Only those studies where the search terms were either in the abstract or the title were considered. The search terms “suicide” OR “suicide prevention” OR “suicide risk” AND “mental health” OR “primary care” in the abstract retrieved over 642 studies across databases, so only those with these search terms in the title were considered. Those dated from 1990 and from all countries were considered, however most published work was from Australia, Europe, New Zealand or USA. This ensured that all studies were included and external validity was improved. Any studies that referred to assisted suicide were excluded, as this involved joint decision making between patient and doctor; and therefore, was not relevant to the research question. All studies found in the scoping review were included in data synthesis, regardless of risk of bias/quality assessment.

### Compliance with Ethical Standards

As this was a scoping review of published data, no ethical approvals were required.

## Results

**Fig. 1:**
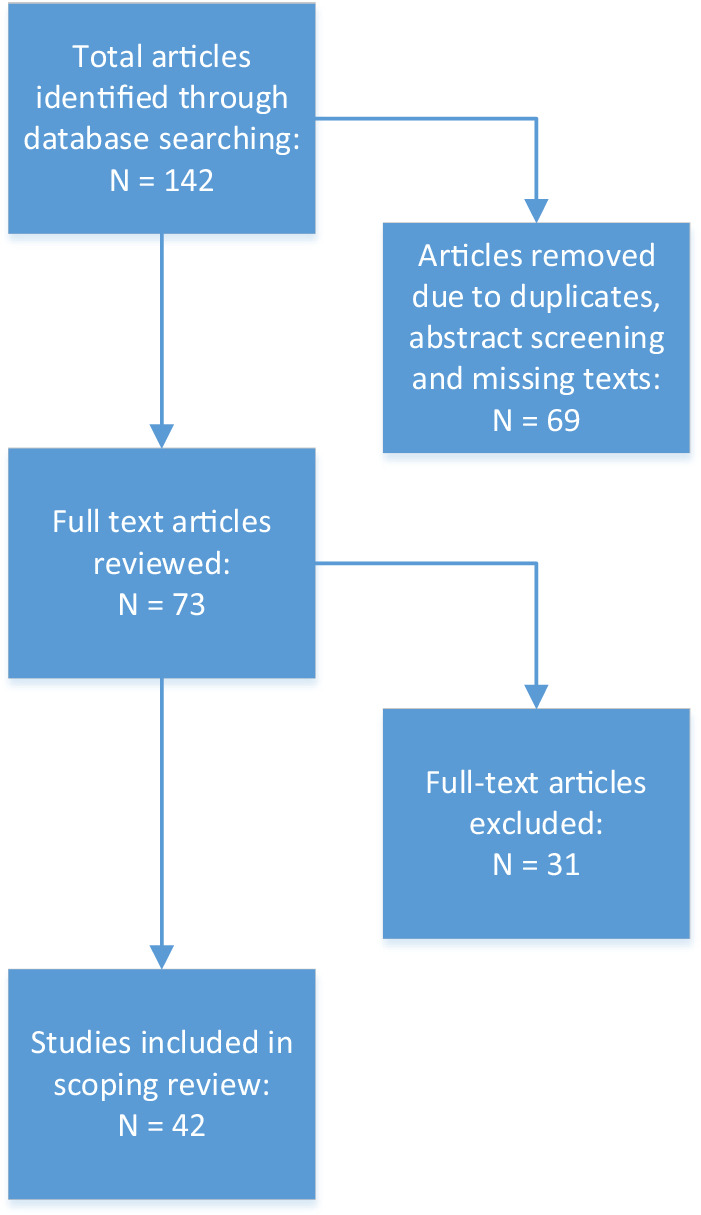
Scoping Search Process

Through both database and manual searches, a total of 142 studies were detected. The titles and abstracts of those studies were screened for significance, resulting in 73 studies being reviewed at full-text level. A further 31 full text papers were excluded due to not focusing on the research question for this scoping review and 42 studies were deemed to meet the inclusion criteria. The process of assessing these studies is displayed in Fig. 1.

An overview of the 42 studies is presented in Tables 2, 3 and 4. The largest proportion of studies primarily used a retrospective analysis of data (n = 14) including reviews of patient case notes and coroner reports. Studies fell into three broad categories (quantitative n = 30, qualitative n = 9, mixed methods n = 3). A typical cross-sectional survey asked about how to identify barriers to effective suicide risk assessment, and a typical experimental study looked at the effectiveness of attitudes to screening for suicidal ideation.

**Table 3:** Qualitative Studies

Author/year/location	Purpose	Participants	Study design	Results
Chandler et al (2015) United Kingdom	To explore how GPs respond to patients who have self-harmed and assess the relationship between Self-Harm, suicide, and suicide risk assessment	30 GPs from Scotland	Semi-structured interviews	There were different conceptualizations across GPs of the relationship between Self-Harm, suicide, and the assessment of suicide risk among GPs
Elzinga et al (2020) Netherlands	To evaluate if the SUPRANET training programme was helpful in supporting PCPs to apply suicide prevention practices	18 Primary Care providers	Semi structured interviews	PCPs discussed the need for better communication with specialist mental health leads. SUPRANET, although helpful, was not enough on its own to improve care
Jerant et al (2019) USA	To develop an understanding of how to encourage men to talk to their PCP about suicidal thoughts	44 adult suicide attempt survivors, family members & Primary Care providers	Semi structured interviews	The fear of immediate hospitalization was identified as a barrier to disclosure
Michail et al. (2016) United Kingdom	To explore GP views and experiences of assessing, communicating with and managing suicidal young people	28 GPs	Semi-structured interviews	Interviews revealed the challenges GPs experience when it comes to the assessment and management of suicide risk in young people and the need for specialist education and training targeting GPs’ knowledge and clinical skills in this field
Muñoz-Sánchez (2018) Spain	To identify key factors for suicide prevention in healthcare	56 healthcare professionals (12 of which primary care physicians)	Focus Group	Primary care physicians identified the need for more time to assess patients for risk and better communication with mental health teams
Perry et al (2020) Australia	To explore young people’s experiences related to the identification and assessment of suicidal behaviour and Self-Harm in Primary Care environment	10 young people aged 16–25	Focus Group	Young people were worried about a loss of privacy when disclosing suicide, were worried about labels such as “risk” and highlighted the importance of GP attitudes
Richards et al (2019) USA	To explore patients experiences of depression screening and suicide risk assessment	37 patients diagnosed with depression	Semi structured interviews	Some participants described a disparity between their lived experience and the PHQ-9 survey. For patients suicidality disclosures involved weighing hope for help against fears of negative consequences
Wainwright et al (2020) United Kingdom	To explore the experiences and needs of parents bereaved by suicide	23 parents bereaved by suicide	Semi structured interviews	Parents had a mixed experience with their GP and highlighted the need for clear communication and signposting to additional support
Wittink et al (2020) USA	To understand the benefits and barriers to a team approach to suicide prevention in primary care for Veterans	67 health care professionals	Semi structured interviews and focus groups	A team approach (nurses, mental health specialists, primary care physicians) can facilitate trust and better outcomes in patients

**Table 4:** Mixed Methods Studies

Author/year/location	Purpose	Participants	Study design	Results
McCabe et al (2017) United Kingdom	Evaluation regarding how professionals interview patients about suicidal ideation in clinical practice	46 Primary Care patients with early diagnosis of depression	Mixed Methods	Closed gateway questions were asked by the GP with the majority of questions creating an expectation of a “no” response and therefore may not elicit a genuine answer
Pearson et al (2009) United Kingdom	To review the nature and frequency of GP visits in the year before suicide	247 patient case notes who died by suicide 159 GP Interviews	Mixed method (retrospective analysis of data and semi structured interviews)	91% of individuals met with their GP in the year prior to their suicide. Agreement between GPs and mental health teams regarding suicide risk was low
Saini et al (2014) United Kingdom	To examine risk assessment and management in primary and secondary care in a clinical sample of individuals who were in contact with mental health services and died by suicide	198 GP interviews 291 patient case notes	Mixed method (retrospective analysis of data and semi structured interviews)	Only 1 in 4 practices had a written suicide protocol and 20% of GPs could not recall any training in the area

Common limitations of the studies included a lack of active control group due to the use of retrospective data and imprecise outcome measures. However this is only a limitation in certain circumstances as it depended on the purpose of the study.

Studies originated from a range of geographical locations including Australia (n = 1), Denmark (n = 1), France (n = 2), Mexico (n = 1), Netherlands (N = 1), Slovenia (n = 2), Spain (n = 1), UK (n = 17), USA (n = 13), and multiple-Europe locations (n = 1). The studies covered a wide range of populations including adult primary care populations (n = 12), GPs and PCP (n = 12), people who had died by suicide (n = 8), a mixture of populations (n = 5), including young people aged 13–24 years (n = 2), older adults aged 60 + years (n = 2) and parents bereaved by suicide (n = 1).

### Key themes

#### Theme 1: Attendance in Primary Care Prior to Suicide

Several studies focussed on patient attendance in primary care prior to death by suicide or suicide attempt. Retrospective data suggested that many patients consulted with their PCP prior to death by suicide or a suicide attempt (Luoma et al., 2002; Pearson et al., 2009; Mesec et al., 2010; Schou et al., 2019). It was observed that females attended for consultations with a physician more frequently, although more males (80%) died by suicide (Haste et al., 1998; Stanistreet et al., 2004). One study found that, when compared with a control group, patients who died by suicide had more interactions with primary care across a ten-year period, but not in the month prior to their death (Power et al., 1997). Young adults (18–39 years) were also less likely to consult with primary care prior to a suicide attempt when compared with older adults (40+ years) (Younes et al., 2013).

#### Theme 2: Reasons for Non-disclosure of Suicidal Behaviour

Four (10%) studies reported barriers and reasons for non-disclosure of suicidal behaviours. The fear of immediate hospitalisation and the impact on perceived masculinity in expressing vulnerability were barriers to men disclosing suicidal thoughts (Jerant et al., 2019). Focus group discussions with young people (aged 16–25) acknowledged loss of privacy when disclosing suicidal thoughts as a barrier to disclosure, as well as the application of labels such as “risk” (Perry et al., 2020). A further study demonstrated context and timing of questions related to self-harm and suicide was important in-patient disclosure, as well as balancing the perceived risk of disclosure (stigma, judgement, hospitalisation) with the benefit of gaining support (Richards et al., 2019). For primary care staff, the lack of time for appointments and assessments was recognised as a key barrier to risk assessment and disclosure (Muñoz-Sánchez et al., 2018).

#### Theme 3: Screening for Suicide Risk

Screening for suicide risk in primary care included using short evidenced-based tools to identify those who may need further evaluation. This may have included assessment of the person's physical condition, previous suicide attempts, alcohol and other drug use, current mental state, history of mental illness, psychosocial factors and determination of current risk of suicide. Evidence showed that primary care staff did not routinely screen all patients for suicidal ideation (Frankenfield et al., 2000; Hooper et al., 2012), and those who were screened were usually noted as ‘low risk’ following assessment (Vannoy et al., 2011), thus highlighting the importance of providing treatment for people of varying levels of risk (Saini et al., 2014). It was recognised that both staff and patients wanted increased routine screening for suicidal thoughts (Bajaj et al., 2008) and that the use of these questions did not have a negative impact on patient’s feelings of self-worth (Crawford et al., 2011).

#### Theme 4: Training for Primary Care Staff

Several studies indicated the need for more training for primary care staff on the utility of suicide risk screening tools alongside interventions and signposting when assessing suicide risk in patients (Frankenfield et al., 2000; Hooper et al., 2012; Saini et al., 2014; Michail et al., 2016; Finnegan et al., 2018). The validity and usefulness of screening tools for suicide risk is continuously debated (Velupillai et al., 2019). To our knowledge, there have been no studies focusing on the impact of suicide prevention practices on long-term patient outcomes in healthcare settings (Gordon et al., 2020). One study on risk assessments in the UK found that there was more emphasis on using suicide risk screening to identify those at risk of suicide than to initiate evidence-based mental health interventions to prevent this outcome (Graney et al., 2020). Suicide risk screening does not therefore reduce suicide attempts when clinical interventions are not implemented (Miller et al., 2017). Although the use of tools such as the Brief Inventory of Thriving (BITS; Binder et al., 2020), Patient Health Questionnaire-2 (PHQ2; Dveweke et al., 2018) and Geriatric Depression Scale (GDS; Heisel et al., 2010), as well as training interventions such as Men and Providers Preventing Suicide (MAPS; Jerant et al., 2020) and Suicide Prevention Action Networks (SUPRANET; Elzinga et al., 2020) can be of benefit to supporting primary care staff when identifying suicidal risk, they need to be used alongside additional approaches (e.g. training for primary care staff, database use to highlight risk factors) and not as the sole approach. Roškar et al. (2010) found an educational programme aimed at primary care physicians on the recognition and treatment of depression and suicide prevention led to an increase of antidepressant prescriptions, but not a reduction in suicides.

#### Theme 5: Use of Language by Primary Care Staff

Several studies recognised the importance of the language used by primary care staff when interacting with patients. It was found that the use of negative closed gateway questions (e.g. “You don’t have thoughts of harming yourself?”) elicited patients to respond that they were not suicidal (McCabe et al., 2017). Similarly, a further study found that in some instances primary care staff unintentionally reinforced the idea of patients not revealing suicidal behaviours through no-problems-expected phrasing (e.g. “You don’t feel suicidal do you?”) (Vannoy et al., 2010). Clear communication was deemed as a key factor in making both patients (Jerant et al., 2020; Vannoy et al., 2010) and parents bereaved by suicide comfortable (Wainwright et al., 2020). Asking evidenced-based questions such as ‘Thoughts that you would be better off dead or of hurting yourself in some way’ from the Patient Health Questionnaire-9 (PHQ-9; Kroenke et al., 2001) have been shown to be reliably accurate in screening individuals with suicidal ideation (Kim et al., 2021).

#### Theme 6: Variation in Suicide Risk Assessment Across Health Services

The approach to the assessment of risk was shown as varying greatly between primary care staff (Chandler et al., 2015) and across different specialities such as mental health leads (Kaplan et al., 1999). Better communication between specialities was identified as a key area for improvement (Elzinga et al., 2020) and that patients had improved outcomes when professionals worked together collaboratively as a team (Wittink et al., 2020). For example, disseminating using clear language and to emphasise media (e.g. multimedia) over text was highlighted (Jerant et al., 2019) as important for communication with patients.

## Discussion

This scoping review included 42 peer-reviewed studies that aimed to explore how PCPs/GPs can effectively recognise and respond to suicide-risk factors in patients consulting in primary care. The important role of primary care in suicide prevention, continues to be highlighted in a range of English national strategies, policies and programmes (DH, ). The research evidence also supported the idea that primary care has a key role in suicide prevention (John et al., 2020; Luoma et al., 2002; Mughal et al., 2021a, 2021b; Pearson et al., 2009) as many people visited their PCP in the months prior to death by suicide and disclosed both distal and proximal risk factors that could help with recognition of those people at risk and provide an opportunity for intervention. Within the review, we identified six key themes regarding suicide risk assessment in primary care. The findings from this research indicated that assessing the probability of dying by suicide has a low positive predictive value and should, therefore, not be the purpose of suicide-screening in primary care. The focus of suicide prevention in primary care should be recognising and responding to suicide-risk factors, such as comorbid physical health diagnosis and substance misuse. Evidence suggested that talking about suicide and responding to suicide-risk factors in primary care did not increase suicidal behaviours but could have prevented them. More training is needed for staff in primary care on assessing risk and communication about suicide behaviours. There are many collaborative efforts to standardise research processes around the world. However, specific components and strategies to include in person-centred suicide risk screening to mitigate suicide and risk in primary care is lacking.

Training was reported as an essential component of primary care for most included studies. Further funds should be allocated for the development of suicide risk screening learning resources and increased training within GP/PCP degree programmes. The lack of consistency in identifying specific training strategies could suggest that GPs/PCPs are at different stages of development based on the type of study being conducted, source of funding, and organisational training requirements. Inconsistencies in training may result in the variation of identifying and treating people at risk of suicide within primary care.

This review highlights the need for GPs/PCPs to be trained on how to identify suicide risk level and to respond accordingly. Strategies that could assist in achieving this would include developing person-centred care that involves the utilisation of validated risk assessment measures to aid communication about suicide. These strategies, however, depend on having knowledgeable and capable practitioners who are trained to discuss suicide more routinely when patients present with evidenced-based risk factors. Strategies tailored to patient needs could be advantageous to ensure patients receive the required treatment suitable to them.

Within the included studies, there was a lack of input from research participants and/or stakeholders described in the use of suicide risk screening tools. For example, there was limited knowledge about the experiences of clinicians utilising these tools within primary care. This is an important consideration for future studies to be participant-oriented and aligned with the patient needs. Working collaborations between patients and clinicians in service redesign has been shown to improve patient and health care outcomes (Bombard et al., 2018). With increased focus on patient engagement in clinical research (Saini et al., 2021), it is imperative to include participant perspectives in the development of suicide prevention initiatives.

### Strengths and Limitations

This scoping review provdes details about the assessment of suicide risk in primary care. It has allowed us to clarify key concepts within the literature and reflect how research is conducted in the area through the inclusion of a wide range of study designs. While this review has several strengths, including the use of an iterative search strategy and a range of databases, there are limitations to note. First, the use of varying terminology to describe suicide risk assessments in the literature may have contributed to the limited number of documents identified for this review. Inconsistent terminology and descriptions can also impede the development of standardised risk assessment tools where there needs to be clarity on processes such as how and when they should be administered. Second, the language of included documents was limited to English; therefore, relevant non-English documents may have been missed. Third**,** this study does not include a formal quality assessment of studies and is not as rigorous as a systematic review. Finally, included studies used a deficit model not an asset model as they describe vulnerabilities or risk factors rather than protective factors.

### Future Implications and Recommendations

Primary care is ideally placed to address the range of suicide-risk factors, including biological, physical-health, psychological and socio-economic factors. Physical-health, psychological and socio-economic factors are the focus of the emerging social prescribing programmes that are being utilised within suicide prevention initiatives (Centre for Mental Health, 2019). This scoping review has highlighted areas for improvement. These include the need for: (1) additional training for primary care staff in suicide prevention; (2) improved communication between partner organisations for improved referrals of patients who communicate suicidal risk; (3) clearer language use by practitioners that allow patients to feel safe to disclose suicidal ideation; and, (4) more intervention based research. Box 1 highlights the key áreas that should be considered when implementing suicide prevention programmes in primary care.

**Box 1:** A suicide prevention programme in primary care needs to consider

The capacity and capability of the whole of the GP/PCP team	
Who and when to screen for suicide-risk factors and how to respond in a timely manner	
How to fully utilise electronic patient records to decide who to screen for suicide risk-factors	
Different suicide-risk-factors for different patient cohorts	
Links with the wider health, social care and community assets; not just specialist mental health services	
How it can adopt and promote a mentally healthy workplace for all its staff; not just GPs	
The needs of those bereaved and affected by suicide including GPs/PCPs	
How to improve the mental health and suicide prevention literacy of patients; particularly those patient groups with higher rates of suicide, such as substance misusers, middle-aged men and those with a history of mental-ill health, self-harm or adverse childhood events	
How to incorporate suicide prevention in the developing social prescribing programmes	

## Conclusion

This scoping review, including peer-reviewed academic literature, provided knowledge for how primary care practitioners can effectively recognise and respond to suicide-risk factors. Previous studies described a range of strategies to address suicide prevention for high-risk groups, assessment of suicide prevention in primary care and evidenced-based suicide prevention interventions, however they have not focused on better recognition and response to specific suicide-risk factors more widely such as poor mental health, substance misuse and long-term physical health conditions. Ideally, these findings could inform the development of person-centred approaches to be used in primary care.

## Data Availability

The data of the paper, which supports the analysis and results of this paper, are available with the corresponding author and the data can be obtained from the authors upon request.
